# Knowledge of Silesia adult inhabitants regarding preventive vaccinations effect on cardiovascular diseases

**DOI:** 10.1186/s12889-022-14337-9

**Published:** 2022-10-20

**Authors:** Józefa Dąbek, Oskar Sierka

**Affiliations:** 1 Department of Cardiology, Faculty of Health Sciences in Katowice, Medical University of Silesia, Ziołowa street 45/47, 40-635 Katowice, Poland; 2 Student Research Group at the Department of Cardiology, Faculty of Health Sciences in Katowice, Medical University of Silesia, Ziołowa street 45/47, 40-635 Katowice, Poland

**Keywords:** Protective vaccinations, Cardiovascular diseases, Adults’ knowledge

## Abstract

**Objectives:**

Protective vaccinations are important in maintaining health and reducing suffering from infectious diseases. Also, vaccine-preventable infectious diseases are associated with the development and progression of cardiovascular diseases.

**Aim:**

The study aimed to test adults' knowledge of the role of protective vaccinations in the prevention of cardiovascular diseases, and their opinions on the quantity of the information provided by doctors in this regard.

**Methods:**

A total of 700 adults participated in the study, most of whom were women (500; 71.43%). The study used an original questionnaire containing questions covering vaccinations and cardiovascular diseases, and the general characteristics of the participants. The inclusion criteria for the study were 18 years of age and written informed consent to participate in the study.

**Results:**

Over 60% of the participants did not know of, or denied the possibility of, developing cardiovascular diseases as a result of avoiding required preventive vaccinations. More than half of the participants stated that there is no need to recommend influenza vaccination to patients with cardiovascular diseases. Over 70% of participants stated that family doctors did not provide sufficient information about protective vaccinations.

**Conclusion:**

In these adults, knowledge of the role of preventive vaccinations in the prevention of cardiovascular diseases was low, and the quantity of the information provided by doctors about preventive vaccinations were considered to be insufficient. Public awareness of the effects of avoiding preventive vaccinations should be raised especially among people with CVD.

**Supplementary Information:**

The online version contains supplementary material available at 10.1186/s12889-022-14337-9.

## Introduction

Protective vaccinations play an extremely important role in maintaining health and reducing the suffering caused by infectious diseases. Infection complications may affect various systems, including the cardiovascular system [1]. Vaccination increases the number of immunized people in a given population, and it decreases the risk of developing disease in those who are not immune, i.e., those who are potentially more susceptible to infection and to a worse course of the disease.

Thanks to the implementation of preventive vaccinations, diseases such as poliomyelitis and smallpox have been eradicated in Poland, and the continuous improvement of the vaccines used and the development of vaccinology make new vaccines safer [2]. Despite the proven effectiveness and safety of their use, there are more and more voices that negatively affect the overall picture of preventive vaccinations.

Vaccine hesitancy continues to develop due to the confluence of social, cultural, political, and personal factors of individual members of society. Vaccine hesitancy is defined by the World Health Organization (WHO) as “delaying the adoption or rejection of safe vaccinations despite their availability” [3, 4].

There is a wide range of reasons for not vaccinating. One of the most significant for many Poles is their mandatory nature [5]. Others include access to unverified information on vaccinations, lack of confidence in their efficacy, previous severe adverse vaccination reactions, and the influence of members of anti-vaccination movements [6].

In recent years, due to the rejection of protective vaccinations, especially in children, the number of cases of previously-rare infectious diseases has been increasing [7]. In Poland, these diseases include, inter alia, measles. According to the National Institute of Hygiene, in 2017 there were 63 cases of measles, and in 2019 already 1,492 cases [8]. The return of infectious diseases undoubtedly contributes to the suffering of patients and the depletion of the already-limited resources of the health care system.

For many years, cardiovascular diseases (CVD) have been the most common cause of death in Poland and the world. In 2021, 92,751 deaths due to cardiovascular diseases were observed in Poland [9]. According to the WHO, 17.9 million people die from cardiovascular diseases each year, which accounts for approximately 32% of deaths worldwide [10]. It is estimated that four out of five deaths from described diseases are caused by heart attacks and strokes. Moreover, one-third of abovementioned deaths occur prematurely, most often in people under the age of 70 [11]. Although genetic factors largely contribute to the development of the diseases in question, environmental and lifestyle factors undoubtedly also play a role in their progression. Some of the commonly known risk factors are smoking, obesity, increased blood cholesterol, low physical activity, and improper nutrition [12].

The role of infectious agents in the development of CVD has been recognized for more than a century [13]. The first scientist who proposed their role in the pathogenesis of CVD was William Osler, who described the relationship between inflammation and atherosclerosis, but the possibility of using vaccinations as a tool in the prevention of CVD was opened by the finding that infection with *Chlamydia pneumoniae* was an independent risk factor for cardiovascular disease, including acute myocardial infarction [14, 15].

Infections, as a stimulus of inflammation, have long been linked to the genesis, progression, and instability of atherosclerotic plaques as well as increased smooth muscle cell proliferation in the vessel wall, which contributes to plaque progression [16]. Various infectious agents inter alia influenza virus have been known to induce local pro-atherosclerotic mechanisms, which include the expression of adhesion molecules and oxidation of low-density lipids. These actions lead to activation of the endothelium, migration of leucocytes, and eventually formation of lipid core [17–19].

Accurate quantitative data on the number of CVD deaths that could be prevented by vaccination are not available in the literature. However, the results of several studies showed the relationship between vaccination and CVD. Influenza and pneumococcal infections may be risk factors for adverse cardiovascular events, especially in high-risk patients [20]. Effective prevention of these CVD risk factors can reduce CVD mortality [21, 22]. In a study by Murphy S. et. al., vaccinations against influenza virus, as secondary prophylaxis, reduced CVD mortality by 55%. Given the impact of influenza vaccination on secondary prevention of CVD death, the 18% vaccination differential between major population subgroups contributes much to disparities in CVD mortality [23]. The 2017 research conducted by Mohseni H et al. showed that among 59,202 patients with heart failure in England between 1990 and 2013, influenza vaccination was associated with a lower risk of hospitalization due to CVD (incidence rate ratio [95% CI] 0.73 [0.71–0.76]), including hospitalizations for heart failure [24]. Corrales-Medina V. et al. found that during first 30 days after hospitalization due to pneumonia caused by *Streptococcus peumoniae* there was a fourfold higher risk of myocardial infarction, which gradually decreased but remained elevated for up to 10 years. In a self-controlled case series of over 22,000 patients conducted by Warren-Gash C. et al., the risk of acute myocardial infarction in the first 3 days after acute respiratory infection due to *S. pneumoniae* exposure in the hospital was significantly increased (incidence rate ratio [95% CI] 4.19 [3.18–5.53]) [25, 26]. After reviewing the above data, it can be concluded that not suffering from or having mild symptoms of a vaccine-preventable disease reduces the risk of developing CVD and, consequently, reduces the likelihood of death from cardiovascular causes.

This study aimed to test adults' knowledge of the role of protective vaccinations in the prevention of cardiovascular diseases, and their opinion on the quantity of the information provided by doctors in this regard.

## Methods

A total of 700 adults participated in the study, most of whom were women (500; 71.43%). The participants were aged from 18 to 83 years, and their mean age was 32.16 ± 16.46 years. The inclusion criteria for the study were 18 years of age, written informed consent to participate in the study, and ability to understand and answer the questions.

All methods used in this study were in line with the applicable guidelines and regulations related to the conduct of scientific research [27, 28]. The presented topic was part of a large project entitled “Medical knowledge of non-professionals as a predictor of health behavior”, which was submitted to the Bioethics Committee at the Medical University of Silesia in Katowice. In their decision of 17.10.2017 (KNW/0022/KB/223/17), members of the Bioethics Committee stated that research based on a survey is not a medical experiment and that neither evaluation nor ethical approval by the committee is required to conduct it. Nevertheless, all participants declared their informed consent to participate in this study.

The study used a proprietary questionnaire prepared in the Polish language. It comprised 3 questions about participants’ knowledge regarding preventive vaccinations’ effect on cardiovascular diseases, 2 about pro-health activities in this regard, 2 about their opinion on the quantity of the information provided by doctors on this subject, 1 intended only for CVD" + " patients regarding recommendations of a cardiologist to perform additional or booster vaccinations, and 6 about the sociodemographic data of the respondents including a question about occurrence of CVD (supplementary material 1). Questionnaire internal consistency was quantified with Cronbach’s alpha coefficient (alpha = 0.76).

Collection of data began in September 2018, and it was completed before the announcement of the global SARS-CoV-2 pandemic in March 2019. Data were collected from inhabitants of the Silesia Voivodeship who were patients of the Department of Cardiology at the Faculty of Health Sciences in Katowice, their visitors, doctors, nurses, and paramedics working in the aforementioned department, and medical students participating in the classes conducted there. Data were also collected from the authors’ acquaintances.

Questionnaires prepared in a traditional paper form were used, and sampling was done using the snowball method. Snowball sampling began when sealed envelopes with the questionnaire inside were handed to participants who had already completed the questionnaire and those participants were kindly requested to provide a few questionnaires to their friends or family members. The authors had no insight into how far the questionnaires had been passed on by the first and subsequent respondents. Completed questionnaires were collected during follow-up visits, and from others by prior arrangement of a convenient date for returning them. The study participants were given a total of 1,200 questionnaires, of which 856 (71.3%) were returned to the authors. Among those 856, 700 had been correctly completed, and their data were analyzed.

Questionnaire completion was completely anonymous and voluntary. The anonymity of the participants was ensured using the following procedures. After completing the questionnaire, each participant placed the completed questionnaire in an attached, white, unmarked envelope, which was sealed by the participant. The sealed envelopes collected from the participants were placed in a specially prepared closed box. The box was opened and questionnaires were removed from the envelopes only when the responses were entered into the database. The procedures described above made it impossible for the researchers to identify the participants.

Descriptive statistics were used to analyze the sociodemographic data, and percentages and distributions for each question on the survey were obtained. All results are presented in figures and tables. The results are divided according to whether the participants reported (CVD" + ") or denied (CVD"-") the presence of cardiovascular disease. Moreover, the results in Supplementary material 2 are divided by age groups, sex, level of education, and the relationship with a profession or medical education. The participants declaring the presence of a relationship with a profession or medical education included doctors, nurses, paramedics, and medical students.

In Tables 1–7 (supplementary material), the first percentage value refers to the percentage calculated in each group (*n* = 166 for CDV" + " and *n* = 534 for CVD"-"), relative to the total value of the given column (placed in the last row of the table), while the value in parentheses refers to the percentage calculated in relation to the sum of the row in individual groups. The percentages calculated for "All participants" express individual numerical values as a percentage of all participants (*n* = 700).

## Results

The general characteristics of the participants, including the presence of cardiovascular diseases, are presented in Table 1.

**Table 1:** General characteristics of the participants, including the presence of cardiovascular diseases

Participants (*n* = 700)							
**Variables**		**CVD” + ”**		**CVD”-”**		**All participants**	
		**n**	**%**	**n**	**%**	**n**	**%**
**Se**x	**Female**	110	15.71	390	55.71	500	71.43
	**Male**	56	8.00	144	20.57	200	28.57
**Age (years)**	**18–30**	26	3.72	328	47.42	358	51.14
	**31–40**	8	1.14	54	7.71	62	8.85
	**41–50**	74	10.57	120	17.14	194	27.71
	**51–60**	14	2.00	14	2.00	28	4.00
	** > 60**	44	6.29	14	2.00	58	8.29
**Average age of participants in a given group**		**50.27 ± 16.52**		**31.37 ± 13.21**		**32.16 ± 16.46**	
**Place of residence**	**City**	148	21.14	464	66.29	612	87.43
	**Village**	18	2.57	70	10.00	88	12.57
**Education**	**Primary**	6	0.86	28	4.00	34	4.86
	**Secondary**	86	12.29	276	39.43	362	51.71
	**Higher**	74	10.57	230	32.86	304	43.43
**Professional or educational connection with medicine**	**Yes**	60	8.57	192	27.43	252	36.00
	**No**	106	15.14	342	48.86	448	64.00
**∑**		**166**	**23.71**	**534**	**76.29**	**700**	**100.00**

**Explanation of abbreviations and symbols: *n*** – number of participants, **CVD" + "** – participants who reported having CVD, **CVD"-"**—participants who denied having CVD, **∑**—sum

Both among the CVD" + " and CVD"-" participants, women dominated. The mean age of the CVD" + " participants was higher than that of the CVD"-" participants. Most of the participants lived in cities. Taking into account the level of education, participants with secondary education constituted the largest group. More than 35% of the participants were people professionally or educationally connected with medicine.

**Fig. 1:**
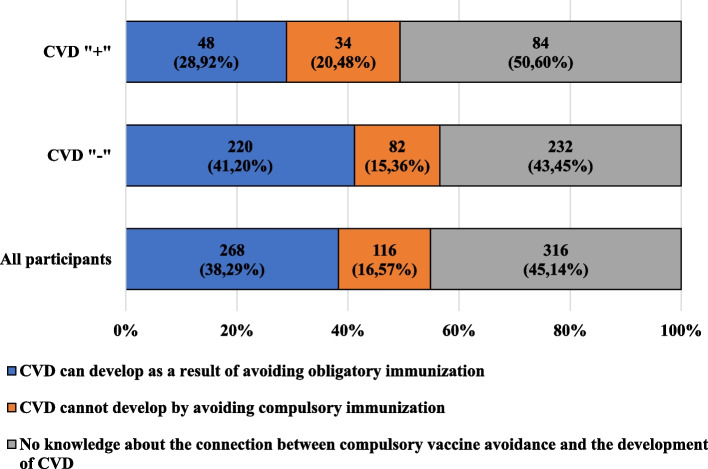
Characteristics of the participants, taking into account their knowledge about the possibility of developing cardiovascular diseases as a result of avoiding obligatory preventive vaccinations and the presence of cardiovascular diseases

Figure 1 presents the characteristics of the participants, taking into account their knowledge about the possibility of developing cardiovascular diseases as a result of avoiding obligatory preventive vaccinations and their occurrence among the participants.

Over 60% of the participants did not know of, or denied the possibility of, developing cardiovascular diseases as a result of avoiding obligatory preventive vaccinations.

More than half of the CVD" + " participants did not have knowledge in this regard, and about 1/5 of them stated that such a relationship does not exist. More men than women, and more people associated professionally or educationally with medicine, compared to unrelated participants, acknowledged that avoiding compulsory immunization may lead to the development of cardiovascular diseases. Among the answers given by the CVD"-" participants, a similar relationship was noted as among CVD" + " participants, taking into account sex and the existence of participants’ professional or educational relationship with medicine.

**Fig. 2:**
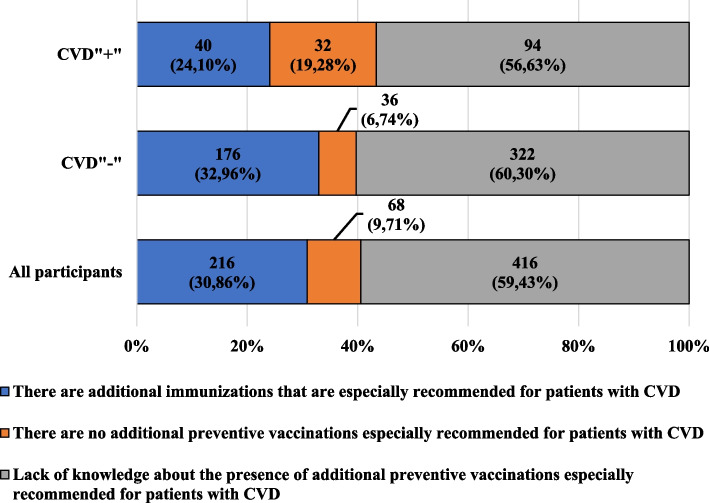
Characteristics of the participants, taking into account their knowledge about additional preventive vaccinations especially recommended for patients with cardiovascular diseases, and presence of cardiovascular diseases

Figure 2 presents the characteristics of the participants, taking into account their knowledge about additional preventive vaccinations, especially recommended for patients with cardiovascular diseases, and their presence in the group of participants.

Only about 31% of all participants knew of additional vaccinations especially recommended for patients with cardiovascular diseases. Over 65% of the CVD"-" participants did not know or denied the existence of additional protective vaccinations, especially recommended for patients with cardiovascular diseases. Both among women and among men, almost half did not know the above-mentioned scope. Regardless of the level of education, the most frequently chosen response to the analyzed question was "I don't know". Only about 1/3 of CVD”- “ participants declaring a connection with medicine acknowledged that vaccinations are especially recommended for patients with cardiovascular diseases. Among the participants with CVD" + ", more than half of women and men did not have knowledge in this area. Only among participants with higher education, there was a greater percentage of responses confirming the presence of the described vaccinations compared to other levels of education, and also more participants who were not professionally or educationally related to medicine, compared to those related, indicated the presence of the above-mentioned type of vaccination.

**Fig. 3:**
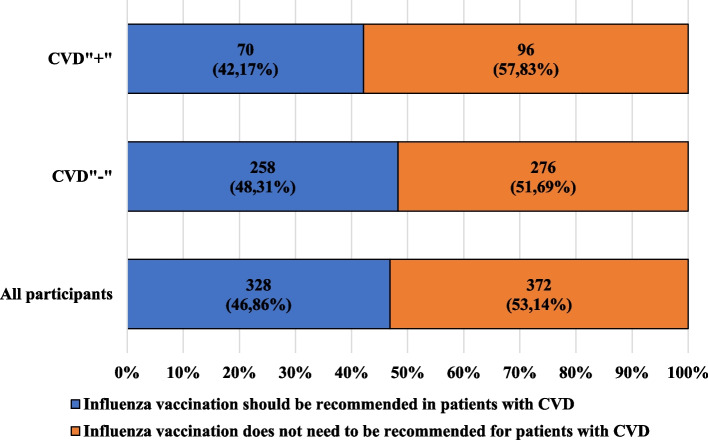
Characteristics of the participants, including their knowledge about the need to recommend influenza vaccination to patients with cardiovascular diseases and presence of cardiovascular diseases

**Fig. 4:**
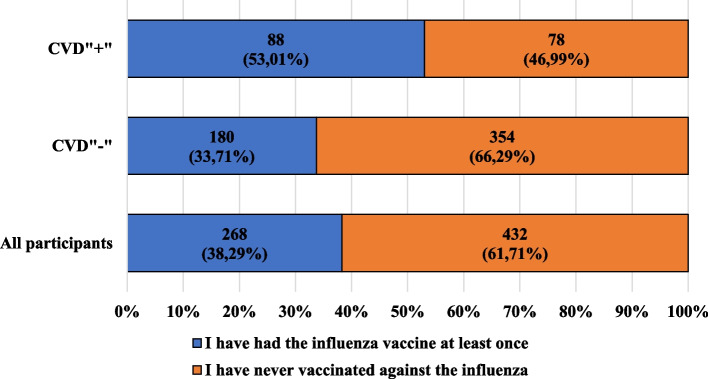
Characteristics of the participants, taking into account administration (at least once) of influenza vaccination and the presence of cardiovascular diseases

**Fig. 5:**
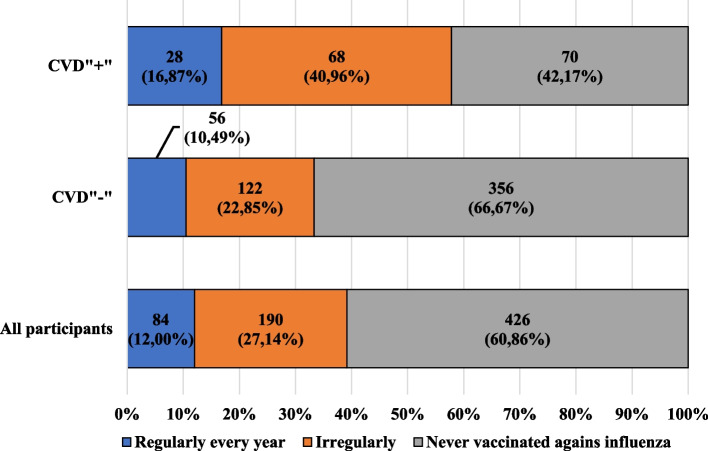
Characteristics of the participants, including regularity of influenza vaccination administration and the presence of cardiovascular diseases

Figure 3 presents the characteristics of the participants, including their knowledge about the need to recommend influenza vaccination to patients with cardiovascular diseases and the presence of these diseases, while Fig. 4 presents the characteristics of the participants, taking into account the admission of (at least once) influenza vaccination and the presence of cardiovascular diseases. Figure 5 presents the characteristics of the participants, taking into account the regularity of influenza vaccination administration and the presence of cardiovascular diseases.

More than half of the participants stated that there is no need to recommend influenza vaccination to patients with cardiovascular diseases, and among CVD" + " the percentage was almost 60%. A similar opinion in the discussed group was shared by 60% of women and over half of the men, moreover, almost 60% of participants from all educational levels denied the above-mentioned necessity.

In the group of CVD"-" participants, half of the men and more than half of the women did not see the need to recommend influenza vaccination to patients with cardiovascular diseases. However, in the 18–30, 31–40, and 51–60 age groups, more participants claimed that the vaccination should be recommended.

Almost 63% of the CVD" + " and about 45% of CVD"-" participants declaring a professional or educational relationship with medicine denied the need to recommend influenza vaccination to the discussed patients.

Almost 40% of participants had never been vaccinated against influenza. In the participants with cardiovascular diseases, vaccination was more often reported by women than by men (60% vs. 39.29%). In the group under discussion, acceptance of the above-mentioned vaccination was reported by the majority of people professionally or educationally connected with medicine.

Among the CVD"-" participants, women were vaccinated against influenza less frequently than men (29.74% vs. 44.44%). In the discussed group, irrespective of their education, almost or slightly more than 60% of participants had not been vaccinated, and among people declaring a professional or educational relationship with medicine, the percentage of people who had not been vaccinated was lower than among those who denied the existence of the described association (63.54% vs. 67.84%).

About 85% of participants were not vaccinated at all or were vaccinated irregularly.

Among the CVD" + " participants, about 3 times more women than men were vaccinated regularly every year. The majority of people being vaccinating each year were people with higher education. Both among those declaring and denying professional or educational connection with medicine, over 40% were not vaccinated against influenza.

Fewer CVD" + " participants were vaccinated annually in relation to the CVD"-" participants and in this group regular, annual vaccination against the virus in question was reported by more men than women (15.28% vs. 8.72%). Among people who were not vaccinated against influenza from the group in question, those with secondary education constituted the highest percentage. Among the CVD"-" participants both reporting and denying the existence of a professional or educational connection with medicine, only about 10% were regularly vaccinated against influenza.

**Fig. 6:**
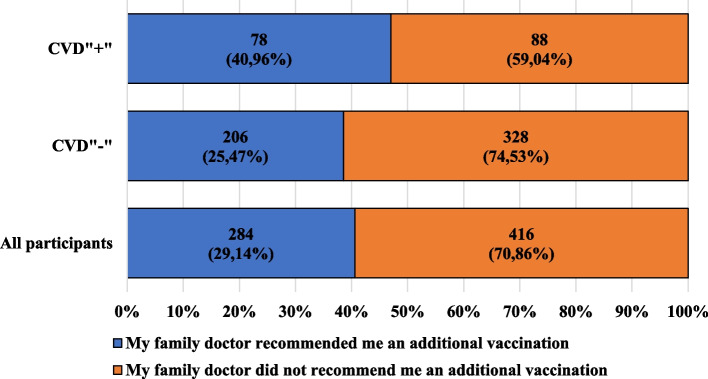
Characteristics of the participants, taking into account the recommendation of the family doctor regarding additional vaccination and presence of cardiovascular diseases

**Fig. 7:**
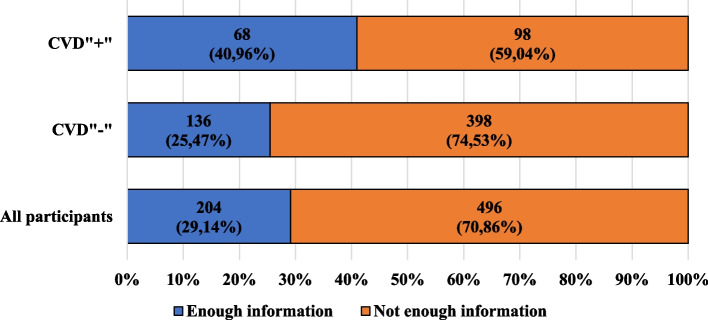
Characteristics of the participants, taking into account their opinions about the quantity of information provided by family doctors regarding protective vaccinations and the presence of cardiovascular diseases

Figure 6 presents the characteristics of the participants, taking into account the recommendation of the family doctor regarding additional vaccination and presence of cardiovascular diseases. Figure 7 shows the characteristics of the participants, taking into account their opinions about the quantity of information provided by family doctors regarding protective vaccinations and the presence of cardiovascular diseases.

In the opinion of over 70% of participants, family doctors do not provide sufficient information on preventive vaccinations. Among the CVD" + " participants, both among women and men, in all age groups, education levels and the presence or absence of a professional or educational relationship with medicine, the majority of participants stated that family doctors do not provide the appropriate amount of information about preventive vaccinations. A similar phenomenon was observed among the CVD"-" participants, with the percentage of people whose family doctor did not recommended additional vaccination being higher among the CVD"-" than CVD" + " participants. As many as 70% of the participants’ family physicians had never offered the recommended vaccination. Among the CVD" + " participants, additional vaccination was offered to a greater percentage of women than men (54.55% vs. 32.14%), and among the CVD"-" participants, additional vaccination was offered to a greater percentage of men than women (44.44% vs. 36.41%).

Table 2 presents the characteristics of the participants with cardiovascular diseases, taking into account the recommendations of a cardiologist to perform additional vaccinations or a booster dose of vaccinations received, and participants’ sex, age, education, and presence of professional or educational relationship with medicine.

**Table 2:** Characteristics of the participants with cardiovascular diseases, taking into account the recommendations of a cardiologist to perform additional or booster vaccinations, as well as sex, age, education, and professional or educational relationship with medicine

Participants with cardiovascular diseases (*n* = 166)						
**Variables**	**CVD**"** + **"				**∑**	
**Possible responses**	**Yes**		**No**		**n**	**%**
	**n**	**%**	**n**	**%**		
**Sex**						
**Males**	6	50,00 (10,71)	50	32,47 (89,29)	**56**	**33,73**
**Females**	6	50,00 (5,45)	104	67,53 (94,55)	**110**	**66,27**
**Age (years)**						
**18–30**	4	33,33 (15,38)	22	14,29 (84,62)	**26**	**15,66**
**31–40**	0	0,0 (0,00)	8	5,19 (100,00)	**8**	**4,82**
**41–50**	8	66,67 (10,81)	66	42,86 (89,19)	**74**	**44,58**
**51–60**	0	0,00 (0,00)	14	9,09 (100,00)	**14**	**8,43**
** > 60**	0	0,00 (0,00)	44	28,57 (100,00)	**44**	**26,51**
**Education**						
**Primary**	0	0,00 (0,00)	6	3,90 (100,00)	**6**	**3,61**
**Secondary**	2	16,67 (2,33)	84	54,55 (97,67)	**86**	**51,81**
**Higher**	10	83,33 (13,51)	64	41,56 (86,49)	**74**	**44,58**
**Professional or educational relationship with medicine**						
**Yes**	4	33,33 (3,77)	102	66,23 (96,23)	**106**	**63,86**
**No**	8	66,67 (13,33)	52	33,77 (86,67)	**60**	**36,14**
**∑**	**12**	**7,23**	**154**	**92,77**	**166**	**100**

**Explanation of abbreviations and symbols: *n***—number of participants, CVD" + " – participants who reported having CVD, ∑—sum

As many as 90% of the CVD" + " participants’ cardiologists had never recommended additional vaccinations or a dose of a booster vaccination. The cardiologists also never recommended the mentioned vaccinations to any participant with primary education.

## Discussion

 The presented results show what a challenge for adults is the topic of the impact of preventive vaccinations on the development of CVD. Among all participants, only about 40% knew about the possibility of developing cardiovascular diseases as a result of avoiding obligatory preventive vaccinations.

In the Polish Vaccination Calendar for 2022, mandatory vaccinations include tuberculosis, hepatitis B, rotavirus, diphtheria, pertussis, tetanus, poliomyelitis, *Haemophilus influenzae* type B, pneumococcus, mumps, measles, and rubella [29].

All of the above pathogens and medical conditions, even if not directly, may contribute to the development of CVD, and the medical literature extensively describes patients suffering from cardiovascular complications of those infectious diseases [30–37].

Interestingly, Kubota Y. et al. postulate a CVD-protective effect of some infections at an early age [38]. The results of their cohort study indicated that higher rates of measles and mumps infections in childhood were associated with a lower risk of death from cardiovascular disease in adulthood. That result led to a "hygiene hypothesis" that more childhood infections result in the production and activation of more regulatory T cells contributing to the suppression of atherosclerosis. Nevertheless, the limitations of that study should not be forgotten. The main one was that the assessment of the number of cases was based on self-assessment of the occurrence of a given disease and that the self-assessment only took into account the exposure to measles and mumps, not taking into account other parameters positively influencing cardiovascular health in Japan [38].

Even though immunization is important in protecting the health of the population, vigorous representatives of anti-vaccination movements downplay their role by drawing attention to the risks associated with vaccine use. Often the alleged "threats" do not actually exist, and what is worse, those heard by people who are unable to make a critical analysis of them shape incorrect attitudes among parents who, by avoiding vaccination, expose their children to serious dangers, and also contribute to the reduction of population immunity [39, 40].

Only about 31% of the participants knew that there are vaccinations especially recommended for patients with cardiovascular diseases. Almost 76% of the participants who knew of the above-mentioned diseases did not have knowledge of, or denied the existence of, additional vaccinations especially recommended for patients with cardiovascular diseases. Moreover, among both CVD" + " and CVD" - " participants, about 55% of those with a professional or educational relationship with medicine denied the need to recommend flu vaccination to patients with cardiovascular diseases.

In agreement with published research, the guidelines of the European Society of Cardiology (ESC) of 2021 on the prevention of cardiovascular diseases state that influenza vaccination can prevent a heart attack caused by the influenza virus [41–43]. The Centers for Disease Control and Prevention (CDC), in addition to recommending vaccination against influenza virus, also recommends vaccination against pneumococci, varicella virus, tetanus, diphtheria, and pertussis (in the form of the Tdap vaccine) [44].

We cannot forget about the need to vaccinate against COVID-19, which can negatively affect the circulatory system. The complications of COVID-19 may include exacerbation of chronic heart failure or the development of cardiomyopathy or myocarditis and venous thromboembolism [45]. The ESC guidelines confirm that patients with myocardial diseases should be vaccinated against SARS-CoV-2 [46]. Almost 40% of the participants in this study had never been vaccinated against influenza, and only 15% had been vaccinated regularly. Importantly, only about 17% of the participants were vaccinated regularly every year. Wójtowicz-Chomicz K. et al. obtained a higher result in their studies: 24.6% of the group of 187 students they studied were regularly vaccinated [47]. On the other hand, Sales I. et al. showed a lower percentage of people who were vaccinated regularly compared to the group from our research. Among 790 residents of Saudi Arabia, approximately 13% were vaccinated regularly against influenza virus [48]. Taking into account the fact that many people with cardiovascular diseases are elderly, an interesting analysis was made by Ye C. et al. They showed that among 4,417 elderly Shanghai residents, only 253 received the influenza vaccine in the 2016/2017 season. Moreover, vaccination was statistically significantly more frequent among elderly people living with their families than among those living alone (*p* < 0.001) [49].

Infection with the influenza virus may lead to systemic inflammatory and thrombogenic responses in the host, causing destabilization of atherosclerotic plaques, hence infection with the influenza virus is associated with an increased risk of cardiovascular events, such as myocardial infarction or stroke, and increased mortality of patients with diseases of the circulatory system [50]. A strong causal relationship between acute respiratory infections and the incidence of cardiovascular events should encourage physicians to recommend vaccination against respiratory infections frequently, and to strongly encourage patients to accept vaccination as a simple prophylactic practice [51].

In the opinion of over 70% of participants, family doctors do not provide enough information on preventive vaccinations, and 70% of participants reported that their family doctors have never offered them the recommended vaccinations or booster vaccinations. What is more, to the CVD" + " participants, cardiologists offered the recommended vaccinations or booster vaccinations only in about 10% of cases. Research on the recommendation of vaccinations by doctors carried out by Bertoldo G. et al. showed that additional vaccination against influenza was not offered to 15% of the 700 adults they studied. On the other hand, the percentage of participants to whom a specialist doctor recommended additional vaccination was 6.4%, which was lower than the percentage obtained in our research [52].

It should be emphasized that members of the health care system are constantly monitored by patients, so it is important how they approach the issue of immunization, especially when it comes to vaccinating their own children.

Research conducted by Kun E. et al. Showed that about 1/3 of family doctors and 50% of community nurses among 765 members of the Hungarian health care system did not see the benefits of immunization [53]. Research by Tomljenovic M. et al. showed that in a group of 465 doctors and nurses, as many as 17% were identified as denying the effectiveness of protective vaccinations [54]. Sternal D. and Owsianko A., in a study of 105 hospital employees, showed that as many as 67.3% of them were not vaccinated against influenza, even though vaccination against influenza is recommended for healthcare professionals [55]. Paris C. et al. studied almost 2,000 employees in the French health care system, and found that when the COVID-19 vaccine appeared, as many as 23% of them hesitated to take it and 4% refused [56]. On the other hand, studies by Karlsson et al. showed that 13% of 2,962 members of the health care system were hesitant before they decided to be vaccinated, 6.3% admitted that they postponed vaccination, and 4.1% rejected compulsory vaccination of their children [57]. The results obtained by Karlsson et al. were similar to those of other studies in this area [58–60].

The limitations of this study include a small group of adults, in which participants without CVD predominated. Moreover, the majority of the participants were young adults, which reduced the number of participants with CVD. Nonetheless, this study gives insight into the knowledge of patients with CVD and people who may take care of such patients, or who may develop cardiovascular diseases themselves. The study also shows whether, according to patients, doctors are providing enough information on immunization to assess the need for change in this area and to introduce new strategies that take into account more diverse understandings of population subgroups.

In summary, regarding the role of preventive vaccinations in the prevention of cardiovascular diseases, the knowledge of adults, both those with and those without cardiovascular diseases, was low. The amount of information provided by doctors on preventive vaccinations and their quality was considered insufficient by the majority of participants. There is a need to raise public awareness of the effects of avoiding preventive vaccinations, especially among members of the health care system, and to mobilize them to provide scientifically valid information in this regard and to recommend vaccinations to their patients. It is important to continuously increase public confidence in this preventive measure and to take all possible measures to effectively combat the demonization of vaccines through social media and other information platforms.

Further research should focus on ways that will ensure the cheapest and most effective way to provide information on immunization in a way that has the greatest impact on society. It is also worth trying to accurately assess the effectiveness of communication between members of the health care system and patients. Data from this study are available from the corresponding author on reasonable request.

## Supplementary Information

**Additional file 1.**

**Additional file 2.**

## Data Availability

The datasets used and analysed in the current study are available from the corresponding author on reasonable request.

## Abbreviations

CVD: Cardiovascular diseases
WHO: World Health Organization

## References

[1] Curry K, Lawson L (2009). Links Between Infectious Diseases and Cardiovascular Disease: A Growing Body of Evidence. J Nurse Pract.

[2] Pieszka M, Waksmańska W, Woś H (2016). Wiedza rodziców dzieci do drugiego roku życia na temat szczepień ochronnych. Med Og Nauk Zdr.

[3] Kestenbaum LA, Feemster KA (2015). Identifying and addressing vaccine hesitancy. Pediatr Ann.

[4] Robertson E, Reeve KS, Niedzwiedz CL, Moor J, Blake M, Green M (2021). Predictors of COVID-19 vaccine hesitancy in the UK household longitudinal study. Brain Behav Immun.

[5] Czajka H (2018). Why are preventive vaccinations still required?. Dev Period Med.

[6] Salmon DA, Dudley MZ, Glanz JM, Omer SB (2015). Vaccine Hesitancy: Causes, Consequences, and a Call to Action. Am J Prev Med..

[7] Hussain A, Ali S, Ahmed M, Hussain S (2019). The Anti-vaccination Movement: A Regression in Modern Medicine. Cureus.

[8] Choroby zakaźne i zatrucia w Polsce w 2020 roku - https://www.gov.pl/web/gis/odra (Accessed: 29.04.2022).

[9] Wojtyniak B, Stokwiszewski J, Rabczenko D, Madej T, Juszczyk G. The mortality of the Polish population according to the cause of death in the first half of 2021 compared with the situation in 2017–2019 and 2020. National Institute of Public Health, Warsaw 2022. https://www.pzh.gov.pl/download/30586/ (Accessed: 29.04.2022).

[10] World Health Organization Cardiovascular Diseases (CVDs)** -**https://www.who.int/news-room/fact-sheets/detail/cardiovascular-diseases-(cvds). (Accessed: 29.04.2022).

[11] World Health Organization - Cardiovascular diseases - https://www.who.int/health-topics/cardiovascular-diseases#tab=tab_1 (Accessed: 29.04.2022).

[12] Marra F, Zhang A, Gillman E, Bessai K, Parhar K, Vadlamudi NK (2020). The protective effect of pneumococcal vaccination on cardiovascular disease in adults: A systematic review and meta-analysis. Int J Infect Dis.

[13] Madjid M, Aboshady I, Awan I, Litovsky S, Casscells SW (2004). Influenza and cardiovascular disease: is there a causal relationship?. Tex Heart Inst J.

[14] Osler W, Fye BF (1985). Lectures on angina pectoris and allied states. William Osler's collected papers on the cardiovascular system.

[15] Dennis J. Cada. Questions and Answers from the F.I.X.. Hosp. Pharm. 2000;35:8, 817–21. 10.3109/07853899909019263.

[16] Pothineni NV, Subramany S, Kuriakose K, Shirazi LF, Romeo F, Shah PK (2017). Infections, atherosclerosis, and coronary heart disease. Eur Heart J.

[17] Haidari M, Wyde PR, Litovsky S, Vela D, Ali M, Casscells SW (2010). Influenza virus directly infects, inflames, and resides in the arteries of atherosclerotic and normal mice. Atherosclerosis.

[18] Park IW, Wang JF, Groopman JE (2001). HIV-1 Tat promotes monocyte chemoattractant protein-1 secretion followed by transmigration of monocytes. Blood.

[19] Li D, Mehta JL (2000). Antisense to LOX-1 inhibits oxidized LDL-mediated upregulation of monocyte chemoattractant protein-1 and monocyte adhesion to human coronary artery endothelial cells. Circulation.

[20] Fountoulaki K, · Tsiodras S, · Polyzogopoulou E, · Olympios C, · Parissis J. Beneficial Effects of Vaccination on Cardiovascular Events: Myocardial Infarction, Stroke, Heart Failure. Cardiology 2018;141:98–106. 10.1159/000493572. 10.1159/000493572 30428464

[21] Mensah GA, Wei GS, Sorlie PD, Fine LJ, Rosenberg Y, Kaufmann PG (2017). Decline in Cardiovascular Mortality: Possible Causes and Implications. Circ Res.

[22] Clar C, Oseni Z, Flowers N, Keshtkar-Jahromi M, Rees K (2015). Influenza vaccines for preventing cardiovascular disease. Cochrane Database Syst Rev.

[23] Murphy SL, Xu J, Kochanek KD. Table 19: Number of deaths, death rates, and age-adjusted death rates for major causes of death: United States, each state, Puerto Rico, Virgin Islands, Guam, American Samoa, and Northern Marianas, 2010. Deaths: Final Data for 2010. Natl Vital Stat Rep. 2013;8;61(4):85–90.

[24] Mohseni H, Kiran A, Khorshidi R, Rahimi K (2017). Influenza vaccination and risk of hospitalization in patients with heart failure: a self-controlled case series study. Eur Heart J.

[25] Corrales-Medina VF, Alvarez KN, Weissfeld LA, Angus DC, Chirinos JA, Chang CC (2015). Association between hospitalization for pneumonia and subsequent risk of cardiovascular disease. JAMA.

[26] Warren-Gash C, Hayward AC, Hemingway H, Denaxas S, Thomas SL, Timmis AD (2012). Influenza infection and risk of acute myocardial infarction in England and Wales: a CALIBER self-controlled case series study. J Infect Dis.

[27] Kelley K, Clark B, Brown V, Sitzia J (2003). Good practice in the conduct and reporting of survey research. Int J Qual Health Care.

[28] Office for Human Research Protections U.S., Office of the Assistan Secretery for Health, Department of Health and Human Services International Compilation of Human Research Standards 2021 Edition - https://www.hhs.gov/sites/default/files/ohrp-international-compilation-2021.pdf (Accessed: 30.08.2022).

[29] Komunikat Głównego Inspektora Sanitarnego z dnia 28 października 2021 r. w sprawie Programu Szczepień Ochronnych na rok 2022 - https://www.gov.pl/attachment/d051a2fe-d74b-478c-b155-05f3671ea1d5 (Accessed: 29.04.2022).

[30] Yaqin W, Jianping X, Meng N, Weiyu X, Ke X, Hongshan Z (2018). Hepatitis B virus and the risk of coronary heart disease: A comprehensive systematic review and meta-analyses of observational studies. Int J of Cardiology.

[31] Michira BN, Alkizim FO, Matheka DM (2015). Patterns and clinical manifestations of tuberculous myocarditis: a systematic review of cases. Pan Afr Med J.

[32] Nakano I, Taniguchi K, Ishibashi-Ueda H, Maeno Y, Yamamoto N, Yui A (2011). Sudden death from systemic rotavirus infection and detection of nonstructural rotavirus proteins. J Clin Microbiol.

[33] Varghese MJ, Ramakrishnan S, Kothari SS, Parashar A, Juneja R, Saxena A (2013). Complete heart block due to diphtheritic myocarditis in the present era. Ann Pediatr Cardiol.

[34] Ledbetter PV, White PD (1925). Effect of pertussis on heart. JAMA.

[35] Araki T, Iwanami N, Yamazaki Y (2019). Severe Tetanus Complicated by Takotsubo Cardiomyopathy. Intern Med.

[36] Melin E, Kahan T, Borg K (2015). Elevated blood lipids are uncommon in patients with post-polio syndrome – a cross-sectional study. BMC Neurol.

[37] Martínez-Quintana E, Rodríguez-González F, Junquera-Rionda P (2012). Congenital rubella syndrome and left pulmonary artery sling. Eur Respir J.

[38] Kubota Y, Iso H, Tamakoshi A (2015). Association of measles and mumps with cardiovascular disease: The Japan Collaborative Cohort (JACC) study. Atherosclerosis.

[39] Field RI (2008). Vaccine declinations present new challenges for public health. P T.

[40] Gundogdu Z (2020). Parental Attitudes and Perceptions Towards Vaccines. Cureus.

[41] Visseren FLJ, Mach F, Smulders YM, Carballo D, Koskinas KC, Bäck M (2021). ESC Guidelines on cardiovascular disease prevention in clinical practice. Eur Heart J.

[42] Udell JA, Zawi R, Bhatt DL, Keshtkar-Jahromi M, Gaughran F, Phrommintikul A (2013). Association between influenza vaccination and cardiovascular outcomes in high-risk patients: a meta-analysis. JAMA.

[43] MacIntyre CR, Mahimbo A, Moa AM, Barnes M (2016). Influenza vaccine as a coronary intervention for prevention of myocardial infarction. Heart.

[44] CDC Vaccine information for adults - https://www.cdc.gov/vaccines/adults/rec-vac/health-conditions/heart-disease.html. (Accessed: 29.04.2022)

[45] Pillai A, Lawson B (2022). Coronavirus disease 2019 and cardiovascular diseases: collateral damage?. Curr Opin Anaesthesiol.

[46] Rosano G, Jankowska EA, Ray R, Metra M, Abdelhamid M, Adamopoulos S (2021). COVID-19 vaccination in patients with heart failure: a position paper of the Heart Failure Association of the European Society of Cardiology. Eur J Heart Fail.

[47] Wójtowicz-Chomicz K, Czeczuk A, Huk-Wieliczuk E, Pikuła A, Borzęcki A (2015). Influenza — flu vaccine or not? Students and knowledge about the flu. Forum Med Rodz.

[48] Sales IA, Syed W, Almutairi MF, Al RY (2021). Public Knowledge, Attitudes, and Practices toward Seasonal Influenza Vaccine in Saudi Arabia: A Cross-Sectional Study. Int J Environ Res Public Health.

[49] Ye C, Zhu W, Yu J, Li Z, Hu W, Hao L (2018). Low coverage rate and awareness of influenza vaccine among older people in Shanghai, China: A cross-sectional study. Hum Vaccin Immunother.

[50] Bhugra P, Grandhi GR, Mszar R, Mszar R, Satish P, Singh R (2021). Determinants of Influenza Vaccine Uptake in Patients with Cardiovascular Disease and Strategies for Improvement. J Am Heart Assoc.

[51] Liprandi ÁS, Liprandi MIS, Zaidel EJ, Aisenberg GM, Baranchuk A, Barbosa ECD (2021). Influenza Vaccination for the Prevention of Cardiovascular Disease in the Americas: Consensus document of the Inter-American Society of Cardiology and the World Heart Federation. Glob Heart.

[52] Bertoldo G, Pesce A, Pepe A, Pelullo CP, Di Giuseppe G (2019). The Collaborative Working Group Seasonal influenza: Knowledge, attitude and vaccine uptake among adults with chronic conditions in Italy. PLoS ONE.

[53] Kun E, Benedek A, Mészner Z (2019). Vaccine hesitancy among primary healthcare professionals in Hungary. Orv Hetil.

[54] Tomljenovic M, Petrovic G, Antoljak N, Hansen L (2021). Vaccination attitudes, beliefs and behaviours among primary health care workers in northern Croatia. Vaccine.

[55] Sternal D, Owsianko A (2019). Opinie personelu medycznego na temat zalecanych szczepień przeciwko grypie Med Og Nauk Zdr.

[56] Paris C, Bénézit F, Geslin M, Polard E, Baldeyrou M, Turmel V (2021). COVID-19 vaccine hesitancy among healthcare workers. Infectious Diseases Now.

[57] Karlsson LC, Lewandowsky S, Antfolk J, Salo P, Lindfelt M, Oksanen T, Kivimäki M, Soveri A. The association between vaccination confidence, vaccination behavior, and willingness to recommend vaccines among Finnish healthcare workers. PLoS One. 2019;14(10):e0224330. 10.1371/journal.pone.0224330. 10.1371/journal.pone.0224330 PMC6822763 31671115

[58] Paterson P, Meurice F, Stanberry LR, Glismann S, Rosenthal SL, Larson HJ (2016). Vaccine hesitancy and healthcare providers. Vaccine.

[59] Filia A, Bella A, D'Ancona F, Fabiani M, Giambi C, Rizzo C, et al. Childhood vaccinations: Knowledge, attitudes and practices of paediatricians and factors associated with their confidence in addressing parental concerns, Italy, 2016. Euro Surveill. 2019;24. 10.2807/1560-7917.ES.2019.24.6.1800275. 10.2807/1560-7917.ES.2019.24.6.1800275 PMC6373065 30755294

[60] Picchio CA, Carrasco MG, Sagué-Vilavella M, Rius C (2019). Knowledge, attitudes and beliefs about vaccination in primary healthcare workers involved in the administration of systematic childhood vaccines, Barcelona, 2016/17. Euro Surveill.

