# Supplementary material for: Knowledge of Silesia adult inhabitants regarding preventive vaccinations effect on cardiovascular diseases
Source: BMC Public Health. 2022 Oct 20;22:1949. doi: 10.1186/s12889-022-14337-9 (PMC9583047; doi:10.1186/s12889-022-14337-9)
Supplement: Supplementary file 1 — Additional file 1. [file 12889_2022_14337_MOESM1_ESM.docx]

**Supplementary material 1**

**Questions asked to the study participants**

**Suppl.1a.** Questions addressed to adult Poles about knowledge of the role of protective vaccinations in the prevention of cardiovascular diseases, and their opinion on the quantity and quality of the information provided by doctors in this regard. Polish version.

| Zadane pytania: |
| --- |
| 1. Proszę podać swój wiek: ……………………………………. |
| 1. Płeć 2. Kobieta 3. Mężczyzna |
| 1. Miejsce zamieszkania   a) Miasto  b) Wieś |
| 1. Please, mark the level of your education; 2. Podstawowe 3. Średnie 4. Wyższe |
| 1. Czy jesteś lekarzem/-arką, pielęgniarzem/-arką, ratowikiem/-iczką, studentem medycyny? 2. Tak 3. Nie |
| 1. Czy leczysz się na schorzenia układu krążenia? 2. Tak 3. Nie |
| 1. Czy unikanie szczepień obowiązkowych może prowadzić do wystąpienia schorzeń ze strony układu krążenia? 2. Tak 3. Nie 4. Nie wiem |
| 1. Czy są jakieś szczepienia szczególnie zalecane u osób ze schorzeniami układu krążenia? 2. Tak 3. Nie 4. Nie wiem |
| 1. Czy pacjentom z chorobami układu krążenia należy zalecać szczepienie przeciwko grypie. 2. Tak 3. Nie |
| 1. Czy kiedykolwiek szczepiłeś/-aś się przeciwko grypie? 2. Tak 3. Nie |
| 1. Jak często szczepisz się przeciwko grypie? 2. Regularnie co roku 3. Nieregularnie 4. Nie szczepię się przeciwko grypie |
| 1. Czy uważasz, że lekarze rodzinni dostarczają wystarczająco informacji na temat szczepień ochronnych? 2. Tak, dostarczają wystarczająco informacji 3. Nie dostarczają wystarczająco informacji |
| 1. Czy kiedykolwiek lekarz rodzinny zaproponował którekolwiek ze szczepień zalecanych? 2. Mój lekarz rodzinny zaproponował mi wykonanie szczepienia dodatkowego 3. Mój lekarz nie zaproponował mi wykonania szczepienia dodatkowego |
| To pytanie jest przeznaczone tylko do respondentów z chorobami układu krążenia.   1. Czy kiedykolwiek kardiolog zalecił, szczepienie zalecane lub dawkę przypominającą szczepienia wcześniej przebytego? 2. Tak 3. Nie |

**Suppl.1b.** Questions addressed to adult Poles about knowledge of the role of protective vaccinations in the prevention of cardiovascular diseases, and their opinion on the quantity and quality of the information provided by doctors in this regard. English version.

| Asked questions*: |
| --- |
| 1. Please, enter your age: ……………………………………. |
| 1. Choose your sex: 2. Female 3. Male |
| 1. Chose your place of residence:   a) City  b) Village |
| 1. Please, mark the level of your education: 2. Primary 3. Secondary 4. Vocational 5. Higher |
| 1. Are you a doctor, nurse, paramedic or medical student? 2. Yes 3. No |
| 1. Are you being treated for cardiovascular diseases? 2. Yes 3. No |
| 1. Can avoiding obligatory preventive vaccinations lead to cardiovascular diseases?   a) Yes  b) No  c) No knowledge |
| 1. Are there any vaccinations especially recommended for people with cardiovascular diseases? 2. Yes 3. No 4. No knowledge |
| 1. Should patients with cardiovascular diseases be advised to vaccinate against influenza. 2. Yes 3. No |
| 1. Have you ever been vaccinated against influenza? 2. Yes 3. No |
| 1. How often do you get vaccinated against the flu? 2. Regularly every year 3. Irregularly 4. I have never been vaccinated against influenza |
| 1. Do you think family doctors provide enough information about protective vaccinations? 2. Yes 3. No |
| 1. Has your family doctor ever proposed any of the recommended vaccinations? a) My family doctor has offered me a recommended vaccination   b) My doctor did not offer me recommended immunization |
| This question is intended only for respondents with cardiovascular disease.   1. Has a cardiologist ever recommended a recommended vaccination or a booster dose for vaccinations before?   a) Yes  b) No |

***Translation made by authors.**
