# Supplementary material for: Knowledge of Silesia adult inhabitants regarding preventive vaccinations effect on cardiovascular diseases
Source: BMC Public Health. 2022 Oct 20;22:1949. doi: 10.1186/s12889-022-14337-9 (PMC9583047; doi:10.1186/s12889-022-14337-9)
Supplement: Supplementary file 2 — Additional file 2. [file 12889_2022_14337_MOESM2_ESM.docx]

**Supplemental material 2**

**Tables with responses**

**Table 1** presents the characteristics of the participants, taking into account the presence of cardiovascular diseases, knowledge of the topic in question, sex, age, level of education, and the participants’ professional or educational relationship with medicine.

**Table 2** presents the characteristics of the participants, taking into account the presence of cardiovascular diseases and their knowledge in the above-mentioned scope, as well as sex, age, education and professional or educational relationship with medicine.

**Table 3** presents the characteristics of the participants, taking into account the presence of cardiovascular diseases, knowledge in the above-mentioned area, sex, age, level of education and professional or educational relationship with medicine.

**Table 4** presents the characteristics of the participants, taking into account the admission (at least once) of influenza vaccination and the presence of cardiovascular diseases, sex, age, level of education and professional or educational relationship with medicine.

**Table 5** presents the characteristics of the participants, including the regularity of taking the above-mentioned vaccination and the occurrence of cardiovascular diseases, sex, age, education and professional or educational relationship of the participants with medicine.

**Table 6** presents the characteristics of the participants, taking into account the opinions on the provision of information by family doctors about vaccinations, the presence of cardiovascular diseases, sex, age, education, and the presence of the participants’ professional or educational connection with medicine.

**Table 7** presents the characteristics of the participants, taking into account the presence of cardiovascular diseases and the vaccination recommended by the family physician, sex, age, education, and professional or educational relationship with medicine.

**Table 1.** Characteristics of the participants, taking into account their knowledge about the possibility of the progression of cardiovascular diseases as a result of avoiding compulsory preventive vaccinations and their occurrence, as well as sex, age, level of education and the presence of a professional or educational connection between the participants and medicine.

| **Participants (n=700; 100%)** | | | | | | | | | | | | | | | | | | | | | | |
| --- | --- | --- | --- | --- | --- | --- | --- | --- | --- | --- | --- | --- | --- | --- | --- | --- | --- | --- | --- | --- | --- | --- |
| **Variables** | **CVD „+”** | | | | | | **∑ n (%)** | **CVD „-”** | | | | | | **∑**  **n (%)** | **Entire studied group** | | | | | | **∑** | |
| **Possible answers** | **Yes** | | **No** | | **I don’t know** | |  | **Yes** | | **No** | | **I don’t know** | |  | **Yes** | | **No** | | **I don’t know** | | **n** | **%** |
|  | **n** | **%** | **n** | **%** | **n** | **%** |  | **n** | **%** | **n** | **%** | **n** | **%** |  | **n** | **%** | **n** | **%** | **n** | **%** |  |  |
| **Sex** | | | | | | | | | | | | | | | | | | | | | | |
| **Males** | 18 | 37,50  (32,14) | 12 | 35,29  (21,43) | 26 | 30,95 (46,43) | **56 (100)** | 68 | 30,91  (47,22) | 24 | 29,27  (16,67) | 52 | 22,41  (36,11) | **144 (100)** | 86 | 12,29 | 36 | 5,14 | 78 | 11,14 | **200** | **28,57** |
| **Females** | 30 | 62,50  (27,27) | 22 | 64,71 (20,00) | 58 | 69,05  (52,73) | **110 (100)** | 152 | 69,09  (38,97) | 58 | 70,73  (14,87) | 180 | 77,59  (46,15) | **390 (100)** | 182 | 26,00 | 80 | 11,43 | 238 | 34,00 | **500** | **71,43** |
| **Age (years)** | | | | | | | | | | | | | | | | | | | | | | |
| **18-30** | 12 | 28,00 (46,15) | 2 | 5,88 (7,69) | 12 | 14,29 (46,15) | **26 (100)** | 174 | 79,09 (52,41) | 52 | 63,42 (15,66) | 106 | 45,69 (31,93) | **332 (100)** | 186 | 26,57 | 54 | 7,71 | 118 | 16,86 | **358** | **51,14** |
| **31-40** | 2 | 4,17  (25,00) | 4 | 11,76  (50,00) | 2 | 2,38  (25,00) | **8 (100)** | 6 | 2,73  (11,11) | 6 | 7,32  (11,11) | 42 | 18,10  (77,78) | **54 (100)** | 8 | 1,14 | 10 | 1,43 | 44 | 6,29 | **62** | **8,86** |
| **41-50** | 20 | 41,67  (27,03) | 20 | 58,82  (27,03) | 34 | 40,48  (45,95) | **74 (100)** | 38 | 17,27  (31,67) | 18 | 21,95  (15,00) | 64 | 27,59  (53,33) | **120 (100)** | 58 | 8,29 | 38 | 5,43 | 98 | 14,00 | **194** | **27,71** |
| **51-60** | 6 | 12,50  (42,86) | 2 | 5,88  (14,29) | 6 | 7,14)  (42,86) | **14 (100)** | 0 | 0,00  (0,00) | 4 | 4,88  (28,57) | 10 | 4,31  (71,43) | **14 (100)** | 6 | 0,86 | 6 | 0,86 | 16 | 2,29 | **28** | **4,00** |
| **>60** | 8 | 16,67  (18,18) | 6 | 17,65  (13,64) | 30 | 35,71  (68,18) | **42 (100)** | 2 | 0,91  (14,29) | 2 | 2,44  (14,29) | 10 | 4,31  (71,43) | **14 (100)** | 10 | 1,43 | 8 | 1,14 | 40 | 5,71 | **58** | **8,29** |
| **Education** | | | | | | | | | | | | | | | | | | | | | | |
| **Primary** | 2 | 4,17  (33,33) | 0 | 0,00  (0,00) | 4 | 4,76  (66,67) | **6 (100)** | 12 | 5,45  (42,86) | 8 | 9,76  (28,57) | 8 | 3,45  (28,57) | **28 (100)** | 14 | 2,00 | 8 | 1,14 | 12 | 1,71 | **34** | **4,86** |
| **Secondary** | 24 | 50,00  (27,91) | 20 | 58,82  (23,26) | 42 | 50,00  (48,84) | **86 (100)** | 136 | 61,82  (49,28) | 42 | 51,22  (15,22) | 98 | 42,24  (35,51) | **276 (100)** | 160 | 22,86 | 62 | 8,86 | 140 | 20,00 | **362** | **51,71** |
| **Higher** | 22 | 45,83  (29,73) | 14 | 41,18  (18,92) | 38 | 45,24  (51,35) | **74 (100)** | 72 | 32,73  (31,30) | 32 | 39,02  (13,91) | 126 | 54,31  (54,78) | **230 (100)** | 94 | 13,43 | 46 | 6,57 | 164 | 23,43 | **304** | **43,43** |
| **Professional or educational relationship with medicine** | | | | | | | | | | | | | | | | | | | | | | |
| **No** | 32 | 66,67  (30,19) | 26 | 76,47  (24,53) | 48 | 57,14  (45,28) | **106 (100)** | 150 | 68,18  (43,86) | 52 | 63,41  (15,20) | 140 | 60,34  (40,94) | **342 (100)** | 182 | 26,00 | 78 | 11,14 | 188 | 26,86 | **448** | **64,00** |
| **Yes** | 16 | 33,33  (26,67) | 8 | 23,53  (13,33) | 36 | 42,86  (60,00) | **60 (100)** | 70 | 31,82  (36,46) | 30 | 36,59  (15,63) | 92 | 39,66  (47,92) | **192 (100)** | 86 | 12,29 | 38 | 5,43 | 128 | 18,29 | **252** | **36,00** |
| **∑** | | | | | | | | | | | | | | | | | | | | | | |
| **∑** | **48** | **28,92** | **34** | **20,48** | **84** | **50,60** | **166 (100)** | **220** | **41,20** | **82** | **15,36** | **232** | **43,45** | **534 (100)** | **268** | **38,29** | **116** | **16,57** | **316** | **45,14** | **700** | **100** |

**Explanation of abbreviations and symbols:** **n** – number of participants, **CVD "+"** - participants who reported having CVD, **CVD "-"** - participants who denied having CVD, **∑** - sum.

**Table 2.** Characteristics of the participants, taking into account their knowledge about additional preventive vaccinations, especially recommended for patients with cardiovascular diseases, and the presence of the aforementioned diseases, sex, age, level of education, and professional or educational relationship of the participants with medicine.

| **Participants (n=700; 100%)** | | | | | | | | | | | | | | | | | | | | | | |
| --- | --- | --- | --- | --- | --- | --- | --- | --- | --- | --- | --- | --- | --- | --- | --- | --- | --- | --- | --- | --- | --- | --- |
| **Variables** | **CVD „+”** | | | | | | **∑**  **n (%)** | **CVD „-”** | | | | | | **∑**  **n (%)** | **Entire studied group** | | | | | | **∑** | |
| **Possible answers** | **Yes** | | **No** | | **No knowledge** | |  | **Yes** | | **No** | | **No knowledge** | |  | **Yes** | | **No** | | **No knowledge** | |  |  |
|  | **n** | **%** | **n** | **%** | **n** | **%** |  | **n** | **%** | **n** | **%** | **n** | **%** |  | **n** | **%** | **n** | **%** | **n** | **%** | **n** | **%** |
| **Sex** | | | | | | | | | | | | | | | | | | | | | | |
| **Males** | 16 | 40,00  (28,57) | 10 | 31,25  (17,86) | 30 | 31,91  (53,57) | **56 (100)** | 60 | 34,09  (41,67) | 14 | 38,89  (9,72) | 70 | 21,74  (48,61) | **144 (100)** | 76 | 10,86 | 76 | 3,43 | 76 | 14,29 | **200** | **28,57** |
| **Females** | 24 | 60,00  (21,82) | 22 | 68,75  (20,00) | 64 | 68,09  (58,18) | **110 (100)** | 116 | 65,91  (29,74) | 22 | 61,11  (5,64) | 252 | 78,26  (64,62) | **390 (100)** | 140 | 20,00 | 44 | 6,29 | 316 | 45,14 | **500** | **71,43** |
| **Age (years)** | | | | | | | | | | | | | | | | | | | | | | |
| **18-30** | 10 | 25,00 (38,46) | 4 | 12,50  (15,38) | 12 | 12,77 (46,15) | **26 (100)** | 124 | 70,45 (37,35) | 20 | 55,56 (6,02) | 188 | 58,39 (56,63) | **332 (100)** | 134 | 19,14 | 24 | 3,43 | 200 | 28,57 | **358** | **51,14** |
| **31-40** | 0 | 0,00)  (0,00) | 2 | 6,25  (25,00) | 6 | 6,38  (75,00) | **8 (100)** | 10 | 5,68  (18,52) | 8 | 22,22  (14,81) | 36 | 11,18  (66,67) | **54 (100)** | 10 | 1,43 | 10 | 1,43 | 42 | 6,00 | **62** | **8,86** |
| **41-50** | 24 | 60,00)  (32,43) | 18 | 32,43  (24,32) | 32 | 34,04  (43,24) | **74 (100)** | 34 | 19,32  (28,33) | 8 | 22,22  (6,67) | 78 | 24,22  (65,00) | **120 (100)** | 58 | 8,29 | 26 | 3,71 | 110 | 15,71 | **194** | **27,71** |
| **51-60** | 2 | 5,00)  (14,29) | 2 | 6,25  (14,29) | 10 | 10,64  (71,43) | **14 (100)** | 6 | 3,41  (42,86) | 0 | 0,00  (0,00) | 8 | 2,48  (57,14) | **14 (100)** | 8 | 1,14 | 2 | 0,29 | 18 | 2,57 | **28** | **4,00** |
| **>60** | 4 | 10,00  (9,09) | 6 | 18,75  (13,64) | 34 | 36,17  (77,27) | **42 (100)** | 2 | 1,14  (14,29) | 0 | 0,00  (0,00) | 12 | 3,73  (85,71) | **14 (100)** | 6 | 0,86 | 6 | 0,86 | 46 | 6,57 | **58** | **8,29** |
| **Education** | | | | | | | | | | | | | | | | | | | | | | |
| **Primary** | 0 | 0,00  (0,00) | 2 | 6,25)  (33,33) | 4 | 4,26  (66,67) | **6 (100)** | 6 | 3,41  (21,43) | 2 | 5,56  (7,14) | 20 | 6,21  (71,43) | **28 (100)** | 6 | 0,86 | 4 | 0,57 | 24 | 3,43 | **34** | **4,86** |
| **Secondary** | 16 | 40,00  (18,60) | 16 | 50,00)  (18,60) | 54 | 57,45  (62,79) | **86 (100)** | 100 | 56,82  (36,23) | 20 | 55,56  (7,25) | 156 | 48,45  (56,52) | **276 (100)** | 116 | 16,57 | 36 | 5,14 | 210 | 30,00 | **362** | **51,71** |
| **Higher** | 24 | 60,00  (32,43) | 14 | 43,75)  (18,92) | 36 | 38,30  (48,65) | **74 (100)** | 72 | 39,77) (30,43) | 14 | 38,89  (6,09) | 146 | 45,34  (63,48) | **230 (100)** | 94 | 13,43 | 28 | 4,00 | 182 | 26,00 | **304** | **43,43** |
| **Professional or educational relationship with medicine** | | | | | | | | | | | | | | | | | | | | | | |
| **No** | 22 | 55,00  (20,75) | 18 | 56,25)  (16,98) | 66 | 70,21  (62,26) | **106 (100)** | 106 | 60,23  (30,99) | 32 | 88,89  (9,36) | 204 | 63,35  (59,65) | **342 (100)** | 128 | 18,29 | 50 | 7,14 | 270 | 38,57 | **448** | **64,00** |
| **Yes** | 18 | 45,00  (30,00) | 14 | 43,75)  (23,33) | 28 | 29,79  (46,67) | **60 (100)** | 70 | 39,77  (36,46) | 4 | 11,11  (2,08) | 118 | 36,65  (61,46) | **192 (100)** | 88 | 12,57 | 18 | 2,57 | 146 | 20,86 | **252** | **36,00** |
| **∑** | | | | | | | | | | | | | | | | | | | | | | |
| **∑** | **40** | **24,10** | **32** | **19,28** | **94** | **56,63** | **166 (100)** | **176** | **32,96** | **36** | **6,74** | **322** | **60,30** | **534 (100)** | **216** | **30,86** | **68** | **9,71** | **416** | **59,43** | **700** | **100** |

**Explanation of abbreviations and symbols:** **n** – number of participants, **CVD "+"** - participants who reported having CVD, **CVD "-"** - participants who denied having CVD, **∑** - sum.

**Table 3.** Characteristics of the participants, taking into account their knowledge about the need to recommend flu vaccination to patients with cardiovascular diseases and the presence of the above-mentioned diseases, sex, age, level of education, and professional or educational relationship with medicine.

| **Participants (n=700; 100%)** | | | | | | | | | | | | | | | | |
| --- | --- | --- | --- | --- | --- | --- | --- | --- | --- | --- | --- | --- | --- | --- | --- | --- |
| **Variables** | **CVD „+”** | | | | **∑**  **n (%)** | **CVD „-”** | | | | **∑**  **n (%)** | **Entire studied group** | | | | **∑** | |
| **Possible answers** | **Yes** | | **No** | |  | **Yes** | | **No** | |  | **Yes** | | **No** | | **n** | **%** |
|  | **n** | **%** | **n** | **%** |  | **n** | **%** | **n** | **%** |  | **n** | **%** | **n** | **%** |  |  |
| **Sex** | | | | | | | | | | | | | | | | |
| **Males** | 26 | 37,14  (46,43) | 30 | 31,25  (53,57) | **56 (100)** | 72 | 27,91  (50,00) | 72 | 26,09  (50,00) | **144 (100)** | 98 | 14,00 | 102 | 14,57 | **200** | **28,57** |
| **Females** | 44 | 62,86  (40,00) | 66 | 68,75  (60,00) | **110 (100)** | 186 | 72,09  (47,69) | 204 | 73,91  (52,31) | **390 (100)** | 230 | 32,86 | 270 | 38,57 | **500** | **71,43** |
| **Age (years)** | | | | | | | | | | | | | | | | |
| **18-30** | 16 | 22,86 (61,54) | 10 | 10,42 (38,46) | **26 (100)** | 172 | 66,66 (51,81) | 160 | 57,97 (48,19) | **332 (100)** | 188 | 26,86 | 170 | 24,28 | **358** | **51,14** |
| **31-40** | 2 | 2,86  (25,00) | 6 | 6,25  (75,00) | **8 (100)** | 34 | 13,18  (62,96) | 20 | 7,25  (37,04) | **54 (100)** | 36 | 5,14 | 26 | 3,71 | **62** | **8,86** |
| **41-50** | 26 | 37,14  (35,14) | 48 | 50,00  (64,86) | **74 (100)** | 42 | 16,28  (35,00) | 78 | 28,26  (65,00) | **120 (100)** | 68 | 9,71 | 126 | 18,00 | **194** | **27,71** |
| **51-60** | 4 | 5,71  (28,57) | 10 | 10,42  (71,43) | **14 (100)** | 8 | 3,10  (57,14) | 6 | 2,17  (42,86) | **14 (100)** | 12 | 1,71 | 16 | 2,29 | **28** | **4,00** |
| **>60** | 22 | 31,43  (50,00) | 22 | 22,92  (50,00) | **42 (100)** | 2 | 0,78  (14,29) | 12 | 4,35  (85,71) | **14 (100)** | 24 | 3,43 | 34 | 4,8 | **58** | **8,29** |
| **Education** | | | | | | | | | | | | | | | | |
| **Primary** | 2 | 2,86  (33,33) | 4 | 4,17  (66,67) | **6 (100)** | 14 | 5,43  (50,00) | 14 | 5,07  (50,00) | **28 (100)** | 16 | 2,29 | 18 | 2,57 | **34** | **4,86** |
| **Secondary** | 38 | 54,29  (44,19) | 48 | 50,00  (55,81) | **86 (100)** | 140 | 54,26  (50,72) | 136 | 49,28  (49,28) | **276 (100)** | 178 | 25,43 | 184 | 26,29 | **362** | **51,71** |
| **Higher** | 30 | 42,86  (40,54) | 44 | 45,83  (59,46) | **74 (100)** | 104 | 40,31  (45,22) | 126 | 45,65  (54,78) | **230 (100)** | 134 | 19,14 | 170 | 24,29 | **304** | **43,43** |
| **Professional or educational relationship with medicine** | | | | | | | | | | | | | | | | |
| **No** | 48 | 68,57  (45,28) | 58 | 60,42  (54,72) | **106 (100)** | 150 | 58,14  (43,86) | 192 | 69,57  (56,14) | **342 (100)** | 198 | 28,29 | 250 | 35,71 | **448** | **64,00** |
| **Yes** | 22 | 45,28  (36,67) | 38 | 39,58  (63,33) | **60 (100)** | 108 | 41,86  (56,25) | 84 | 30,43  (43,75) | **192 (100)** | 130 | 18,57 | 122 | 17,43 | **252** | **36,00** |
| **∑** | | | | | | | | | | | | | | | | |
| **∑** | **70** | **42,17** | **96** | **57,83** | **166 (100)** | **258** | **48,31** | **276** | **51,69** | **534  (100)** | **328** | **46,86** | **372** | **53,14** | **700** | **100** |

**Explanation of abbreviations and symbols:** **n** – number of participants, **CVD "+"** - participants who reported having CVD, **CVD "-"** - participants who denied having CVD, **∑** - sum.

**Table 4.** Characteristics of the participants, taking into account (at least once) vaccination against influenza and the presence of cardiovascular diseases, sex, age, level of education, and the presence of a professional or educational connection between the participants and medicine.

| **Participants (n=700; 100%)** | | | | | | | | | | | | | | | | |
| --- | --- | --- | --- | --- | --- | --- | --- | --- | --- | --- | --- | --- | --- | --- | --- | --- |
| **Variables** | **CVD „+”** | | | | **∑**  **n (%)** | **CVD „-”** | | | | **∑**  **n (%)** | **Entire studied group** | | | | **∑** | |
| **Possible answers** | **Yes** | | **No** | |  | **Yes** | | **No** | |  | **Yes** | | **No** | | **n** | **%** |
|  | **n** | **%** | **n** | **%** |  | **n** | **%** | **n** | **%** |  | **n** | **%** | **n** | **%** |  |  |
| **Sex** | | | | | | | | | | | | | | | | |
| **Males** | 22 | 25,00  (39,29) | 34 | 43,59  (60,71) | **56 (100)** | 64 | 35,56  (44,44) | 80 | 22,60  (55,56) | **144 (100)** | 86 | 12,29 | 114 | 16,29 | **200** | **28,57** |
| **Females** | 66 | 75,00  (60,00) | 44 | 56,41  (40,00) | **110 (100)** | 116 | 64,44  (29,74) | 274 | 77,40  (70,26) | **390 (100)** | 182 | 26,00 | 318 | 45,43 | **500** | **71,43** |
| **Age (years)** | | | | | | | | | | | | | | | | |
| **18-30** | 8 | 9,09 (30,77) | 18 | 23,07 (69,23%) | **26 (100)** | 114 | 63,33  (34,34) | 218 | 61,58 (65,55) | **332 (100)** | 122 | 17,43 | 236 | 33,71 | 358 | 51,14 |
| **31-40** | 2 | 2,27  (25,00) | 6 | 7,69  (75,00) | **8 (100)** | 10 | 5,56  (18,52) | 44 | 12,43  (81,48) | **54 (100)** | 12 | 1,71 | 50 | 7,14 | **62** | **8,86** |
| **41-50** | 52 | 59,09  (70,27) | 22 | 28,21  (29,73) | **74 (100)** | 50 | 27,78  (41,67) | 70 | 19,77  (58,33) | **120 (100)** | 102 | 14,57 | 92 | 13,14 | **194** | **27,71** |
| **51-60** | 6 | 6,82  (42,86) | 8 | 10,26  (57,14) | **14 (100)** | 4 | 2,22  (28,57) | 10 | 2,82  (71,43) | **14 (100)** | 10 | 1,43 | 18 | 2,57 | **28** | **4,00** |
| **>60** | 20 | 22,73  (45,45) | 24 | 30,77  (54,55) | **42 (100)** | 2 | 1,11  (14,29) | 12 | 3,39  (85,71) | **14 (100)** | 22 | 3,14 | 36 | 5,14 | **58** | **8,29** |
| **Education** | | | | | | | | | | | | | | | | |
| **Primary** | 4 | 4,55  (66,67) | 2 | 2,56  (33,33) | **6 (100)** | 12 | 6,67  (42,86) | 16 | 4,52  (57,14) | **28 (100)** | 16 | 2,29 | 18 | 2,57 | **34** | **4,86** |
| **Secondary** | 36 | 40,91  (41,86) | 50 | 64,10  (58,14) | **86 (100)** | 86 | 47,78  (31,16) | 190 | 53,67  (68,84) | **276 (100)** | 122 | 17,43 | 240 | 34,29 | **362** | **51,71** |
| **Higher** | 48 | 54,55  (64,86) | 26 | 33,33  (35,14) | **74 (100)** | 82 | 45,56  (35,65) | 148 | 41,81  (64,35) | **230 (100)** | 130 | 18,57 | 174 | 24,86 | **304** | **43,43** |
| **Professional or educational relationship with medicine** | | | | | | | | | | | | | | | | |
| **No** | 64 | 72,73  (60,38) | 42 | 53,85  (39,62) | **106 (100)** | 110 | 61,11  (32,16) | 232 | 65,54  (67,84) | **342 (100)** | 174 | 24,86 | 274 | 39,14 | **448** | **64,00** |
| **Yes** | 24 | 27,27  (40,00) | 36 | 46,15  (60,00) | **60 (100)** | 70 | 38,89  (36,46) | 122 | 34,46  (63,54) | **192 (100)** | 94 | 13,43 | 158 | 22,57 | **252** | **36,00** |
| **∑** | | | | | | | | | | | | | | | | |
| **∑** | **88** | **53,01** | **78** | **46,99** | **166**  **(100)** | **180** | **33,71** | **354** | **66,29** | **534 (100)** | **268** | **38,29** | **432** | **61,71** | **700** | **100** |

**Explanation of abbreviations and symbols:** **n** – number of participants, **CVD "+"** - participants who reported having CVD, **CVD "-"** - participants who denied having CVD, **∑** - sum.

**Table 5.** Characteristics of the participants, taking into account the regularity of receiving flu vaccinations and the presence of cardiovascular diseases, sex, age, education, and the presence of a professional or educational connection with medicine.

| **Participants (n=700; 100%)** | | | | | | | | | | | | | | | | | | | | | | |
| --- | --- | --- | --- | --- | --- | --- | --- | --- | --- | --- | --- | --- | --- | --- | --- | --- | --- | --- | --- | --- | --- | --- |
| **Variables** | **CVD „+”** | | | | | | **∑**  **n (%)** | **CVD „-”** | | | | | | **∑**  **n (%)** | **Entire studied group** | | | | | | **∑** | |
| **Possible answers** | **Regularly every year** | | **Irregularly** | | **No vaccinations against influenza** | |  | **Regularly every year** | | **Irregularly** | | **No vaccinations against influenza** | |  | **Regularly every year** | | **Irregularly** | | **No vaccinations against influenza** | | **n** | **%** |
|  | **n** | **%** | **n** | **%** | **n** | **%** |  | **n** | **%** | **n** | **%** | **n** | **%** |  | **n** | **%** | **n** | **%** | **n** | **%** |  |  |
| **Sex** | | | | | | | | | | | | | | | | | | | | | | |
| **Males** | 4 | 14,29  (7,14) | 22 | 32,35  (39,29) | 30 | 42,86  (53,57) | **56 (100)** | 22 | 39,29  (15,28) | 48 | 39,34  (33,33) | 74 | 20,79  (51,39) | **144 (100)** | 26 | 3,7 | 70 | 10,00 | 104 | 14,86 | **200** | **28,57** |
| **Females** | 24 | 85,71  (21,82) | 46 | 67,65  (41,82) | 40 | 57,14  (36,36) | **110 (100)** | 34 | 60,71  (8,72) | 74 | 60,66  (18,97) | 282 | 79,21  (72,31) | **390 (100)** | 58 | 8,29 | 120 | 17,14 | 322 | 46,00 | **500** | **71,43** |
| **Age (years)** | | | | | | | | | | | | | | | | | | | | | | |
| **18-30** | 4 | 14,29 (15,38) | 6 | 8,82 (23,08) | 16 | 22,86 (61,54) | **26 (100)** | 34 | 60,72 (10,24) | 80 | 65,58 (24,10) | 218 | 61,24 (65,66) | **332 (100)** | 38 | 5,42 | 86 | 12,28 | 234 | 29,43 | 358 | 51,14 |
| **31-40** | 0 | 0,00  (0,00) | 4 | 5,88  (50,00) | 4 | 5,71  (50,00) | **8 (100)** | 2 | 3,57  (3,70) | 6 | 4,92  (11,11) | 46 | 12,92  (85,19) | **54 (100)** | 2 | 0,29 | 10 | 1,43 | 50 | 7,14 | **62** | **8,86** |
| **41-50** | 14 | 50,00  (18,92) | 36 | 52,94  (48,65) | 24 | 34,29  (32,43) | **74 (100)** | 16 | 28,57  (13,33) | 34 | 27,87  (28,33) | 70 | 19,66  (58,33) | **120 (100)** | 30 | 4,29 | 70 | 10,00 | 94 | 13,43 | **194** | **27,71** |
| **51-60** | 4 | 14,29  (28,57) | 2 | 2,94  (14,29) | 8 | 11,43  (57,14) | **14 (100)** | 2 | 3,57  (14,29) | 2 | 1,64  (14,29) | 10 | 2,81  (71,43) | **14 (100)** | 6 | 0,86 | 4 | 0,57 | 18 | 2,57 | **28** | **4,00** |
| **>60** | 6 | 21,43  (13,64) | 20 | 29,41  (45,45) | 18 | 25,71  (40,91) | **42 (100)** | 2 | 3,57  (14,29) | 0 | 0,00  (0,00) | 12 | 3,37  (85,71) | **14 (100)** | 8 | 1,14 | 20 | 2,8 | 30 | 4,29 | **58** | **8,29** |
| **Education** | | | | | | | | | | | | | | | | | | | | | | |
| **Primary** | 2 | 7,14  (33,33) | 2 | 2,94  (33,33) | 2 | 2,86  (33,33) | **6 (100)** | 6 | 10,71  (21,43) | 12 | 9,84  (42,86) | 10 | 2,81  (35,71) | **28 (100)** | 8 | 1,14 | 14 | 2,00 | 12 | 1,71 | **34** | **4,86** |
| **Secondary** | 8 | 28,57  (9,30) | 38 | 55,88  (44,19) | 40 | 57,14  (46,51) | **86 (100)** | 2 | 39,29  (7,97) | 60 | 49,18  (21,74) | 194 | 54,49  (70,29) | **276 (100)** | 30 | 4,29 | 98 | 14,00 | 234 | 33,43 | **362** | **51,71** |
| **Higher** | 18 | 64,29  (24,32) | 28 | 41,18  (37,84) | 28 | 40,00  (37,84) | **74 (100)** | 28 | 50,00  (12,17) | 50 | 40,98  (21,74) | 152 | 42,70  (66,09) | **230 (100)** | 46 | 6,57 | 78 | 11,14 | 180 | 25,71 | **304** | **43,43** |
| **Professional or educational relationship with medicine** | | | | | | | | | | | | | | | | | | | | | | |
| **No** | 18 | 64,29  (16,98) | 44 | 64,71  (41,51) | 44 | 62,86  (41,51) | **106 (100)** | 36 | 64,29  (10,53) | 76 | 62,30  (22,22) | 230 | 64,61  (67,25) | **342 (100)** | 54 | 7,71 | 120 | 17,14 | 274 | 39,14 | **448** | **64,00** |
| **Yes** | 10 | 35,71  (16,67) | 24 | 35,29  (40,00) | 26 | 37,14  (43,33) | **60 (100)** | 20 | 35,71  (10,42) | 46 | 37,70  (23,96) | 126 | 35,39  (65,63) | **192 (100)** | 30 | 4,29 | 70 | 10,00 | 152 | 21,71 | **252** | **36,00** |
| **∑** | | | | | | | | | | | | | | | | | | | | | | |
| **∑** | **28** | **16,87** | **68** | **40,96** | **70** | **42,17** | **166 (100)** | **56** | **10,49** | **122** | **22,85** | **356** | **66,67** | **534 (100) _** | **84** | **12,00** | **190** | **27,14** | **426** | **60,86** | **700** | **100** |

**Explanation of abbreviations and symbols:** **n** – number of participants, **CVD "+"** - participants who reported having CVD, **CVD "-"** - participants who denied having CVD, **∑** - sum.

**Table 6.** Characteristics of the participants, taking into account the opinions on the amount of information provided by family doctors about preventive vaccinations and the occurrence of cardiovascular diseases, sex, age, education, and professional or educational relationship with medicine.

| **Participants (n=700; 100%)** | | | | | | | | | | | | | | | | |
| --- | --- | --- | --- | --- | --- | --- | --- | --- | --- | --- | --- | --- | --- | --- | --- | --- |
| **Variables** | **CVD „+”** | | | | **∑**  **n (%)** | **CVD „-”** | | | | **∑**  **n (%)** | **Entire studied group** | | | | **∑** | |
| **Possible answers** | **Enough information** | | **Not enough information** | |  | **Enough information** | | **Not enough information** | |  | **Enough information** | | **Not enough information** | | **n** | **%** |
|  | **n** | **%** | **n** | **%** |  | **n** | **%** | **n** | **%** |  | **n** | **%** | **n** | **%** |  |  |
| **Sex** | | | | | | | | | | | | | | | | |
| **Males** | 20 | 29,41  (35,71) | 36 | 36,73  (64,29) | **56 (100)** | 42 | 30,88  (29,17) | 102 | 25,63  (70,83) | **144 (100)** | 62 | 8,86 | 138 | 19,71 | **200** | **28,57** |
| **Females** | 48 | 70,59  (43,64) | 62 | 63,27  (56,36) | **110 (100)** | 94 | 69,12  (24,10) | 296 | 74,37  (75,90) | **390 (100)** | 142 | 20,29 | 358 | 51,14 | **500** | **71,43** |
| **Age (years)** | | | | | | | | | | | | | | | | |
| **18-25** | 8 | 11,76 (30,77) | 18 | 18,37 (69,23) | **26 (100)** | 74 | 54,41 (22,29) | 258 | 64,82 (77,71) | **332 (100)** | 82 | 11,71 | 276 | 39,43 | 358 | 51,14 |
| **31-40** | 2 | 2,94  (25,00) | 6 | 6,12  (75,00) | **8 (100)** | 16 | 11,76  (29,63) | 38 | 9,55  (70,37) | **54 (100)** | 18 | 2,57 | 44 | 6,29 | **62** | **8,86** |
| **41-50** | 34 | 50,00  (45,95) | 40 | 40,82  (54,05) | **74 (100)** | 32 | 23,53  (26,67) | 88 | 22,11  (73,33) | **120 (100)** | 66 | 9,4 | 128 | 18,29 | **194** | **27,71** |
| **51-60** | 6 | 8,82  (42,86) | 8 | 8,16  (57,14) | **14 (100)** | 8 | 5,88  (57,14) | 6 | 1,51  (42,86) | **14 (100)** | 14 | 2,00 | 14 | 2,0 | **28** | **4,00** |
| **>60** | 18 | 26,47  (40,91) | 26 | 26,53  (59,09) | **42 (100)** | 6 | 4,41  (42,86) | 8 | 2,01  (57,14) | **14 (100)** | 24 | 3,43 | 34 | 4,86 | **58** | **8,29** |
| **Education** | | | | | | | | | | | | | | | | |
| **Primary** | 2 | 2,94  (33,33) | 4 | 4,08  (66,67) | **6 (100)** | 6 | 4,41  (21,43) | 22 | 5,53  (78,57) | **28 (100)** | 8 | 1,14 | 26 | 3,71 | **34** | **4,86** |
| **Secondary** | 44 | 64,71  (51,16) | 42 | 42,86  (48,84) | **86 (100)** | 62 | 45,59  (22,46) | 214 | 53,77  (77,54) | **276 (100)** | 106 | 15,14 | 256 | 36,57 | **362** | **51,71** |
| **Higher** | 22 | 32,35  (29,73) | 52 | 53,06  (70,27) | **74 (100)** | 68 | 50,00  (29,57) | 162 | 40,70  (70,43) | **230 (100)** | 90 | 12,86 | 214 | 30,57 | **304** | **43,43** |
| **Professional or educational relationship with medicine** | | | | | | | | | | | | | | | | |
| **No** | 46 | 67,65  (43,40) | 60 | 61,22  (56,60) | **106 (100)** | 102 | 75,00  (29,82) | 240 | 60,30  (70,18) | **342 (100)** | 148 | 21,14 | 300 | 42,86 | **448** | **64,00** |
| **Yes** | 22 | 32,35  (36,67) | 38 | 38,78  (63,33) | **60 (100)** | 34 | 25,00  (17,71) | 158 | 39,70  (82,29) | **192 (100)** | 56 | 8,00 | 196 | 28,00 | **252** | **36,00** |
| **∑** | | | | | | | | | | | | | | | | |
| **∑** | **68** | **40,96** | **98** | **59,04** | **166 (100)** | **136** | **25,47** | **398** | **74,53** | **534 (100)** | 204 | 29,14 | 496 | 70,86 | **700** | **100** |

**Explanation of abbreviations and symbols:** **n** – number of participants, **CVD "+"** - participants who reported having CVD, **CVD "-"** - participants who denied having CVD, **∑** - sum.

**Table 7.** Characteristics of the participants, taking into account the vaccination recommended by the family doctor to the participants and the presence of cardiovascular diseases, sex, age, education, and professional or educational relationship with medicine.

| **Participants (n=700; 100%)** | | | | | | | | | | | | | | | | |
| --- | --- | --- | --- | --- | --- | --- | --- | --- | --- | --- | --- | --- | --- | --- | --- | --- |
| **Variables** | **CVD „+”** | | | | **∑  n (%)** | **CVD „-”** | | | | **∑ n (%)** | **Entire studied group** | | | | **∑** | |
| **Possible answers** | **Yes** | | **No** | |  | **Yes** | | **No** | |  | **Yes** | | **No** | | **n** | **%** |
|  | **n** | **%** | **n** | **%** |  | **n** | **%** | **n** | **%** |  | **n** | **%** | **n** | **%** |  |  |
| **Sex** | | | | | | | | | | | | | | | | |
| **Males** | 18 | 23,08  (32,14) | 38 | 43,18  (67,86) | **56 (100)** | 64 | 31,07  (44,44) | 80 | 24,39  (55,56) | **144 (100)** | 82 | 11,71 | 11 | 16,86 | **200** | **28,57** |
| **Females** | 60 | 76,92  (54,55) | 50 | 56,82  (45,45) | **110 (100)** | 142 | 68,93  (36,41) | 248 | 75,61  (63,59) | **390 (100)** | 202 | 28,86 | 29 | 42,57 | **500** | **71,43** |
| **Age (years)** | | | | | | | | | | | | | | | | |
| **18-30** | 10 | 12,82  (38,46) | 16 | 18,18 (61,54) | **26 (100)** | 108 | 52,43 (32,53) | 224 | 68,30 (67,47) | **332 (100)** | 118 | 16,86 | 240 | 34,28 | 358 | 51,14 |
| **31-40** | 4 | 5,13  (50,00) | 4 | 4,55  (50,00) | **8 (100)** | 26 | 12,62  (48,15) | 28 | 8,54  (51,85) | **54 (100)** | 30 | 4,2 | 32 | 4,57 | **62** | **8,86** |
| **41-50** | 42 | 53,85  (56,76) | 32 | 36,36  (43,24) | **74 (100)** | 52 | 25,24  (43,33) | 68 | 20,73  (56,67) | **120 (100)** | 94 | 13,43 | 100 | 14,29 | **194** | **27,71** |
| **51-60** | 6 | 7,69  (42,86) | 8 | 9,09  (57,14) | **14 (100)** | 10 | 4,85  (71,43) | 4 | 1,22  (28,57) | **14 (100)** | 16 | 2,29 | 12 | 1,7 | **28** | **4,00** |
| **>60** | 16 | 57,14  (36,36) | 28 | 31,82  (63,64) | **42 (100)** | 10 | 4,85  (71,43) | 4 | 1,22  (28,57) | **14 (100)** | 26 | 3,71 | 32 | 4,57 | **58** | **8,29** |
| **Education** | | | | | | | | | | | | | | | | |
| **Primary** | 0 | 0,00  (0,00) | 6 | 6,82  (100,00 | **6 (100)** | 14 | 6,80  (50,00) | 14 | 4,27  (50,00) | **28 (100)** | 14 | 2,00 | 20 | 2,86 | **34** | **4,86** |
| **Secondary** | 30 | 38,46  (34,88) | 56 | 63,64  (65,12) | **86 (100)** | 94 | 45,63  (34,06) | 182 | 55,49  (65,94) | **276 (100)** | 124 | 17,71 | 238 | 34,00 | **362** | **51,71** |
| **Higher** | 48 | 61,54  (64,86) | 26 | 29,55  (35,14) | **74 (100)** | 98 | 47,57  (42,61) | 132 | 40,24  (57,39) | **230 (100)** | 146 | 20,86 | 158 | 22,57 | **304** | **43,43** |
| **Professional or educational relationship with medicine** | | | | | | | | | | | | | | | | |
| **No** | 44 | 56,41  (41,51) | 62 | 70,45  (58,49) | **106 (100)** | 140 | 67,96  (40,94) | 202 | 61,59  (59,06) | **342 (100)** | 184 | 26,29 | 264 | 37,71 | **448** | **64,00** |
| **Yes** | 34 | 43,59  (56,67) | 26 | 29,55  (43,33) | **60 (100)** | 66 | 32,04  (34,38) | 126 | 38,41  (65,63) | **192 (100)** | 100 | 14,29 | 152 | 21,71 | **252** | **36,00** |
| **∑** | | | | | | | | | | | | | | | | |
| **∑** | **78** | **46,99** | **88** | **53,01** | **166 (100)** | **206** | **38,58** | **328** | **61,42** | **534 (100)** | **284** | **40,57** | **416** | **59,43** | **700** | **100** |

**Explanation of abbreviations and symbols:** **n** – number of participants, **CVD "+"** - participants who reported having CVD, **CVD "-"** - participants who denied having CVD, **∑** - sum.
